# Maternal Cell-Free DNA Analysis in a Fetus Affected by Beckwith-Wiedemann Syndrome: Potential for Prenatal Diagnosis

**DOI:** 10.7759/cureus.82215

**Published:** 2025-04-13

**Authors:** Ryutaro Yamamoto, Takeshi Umazume, Hiroshi Asano, Satoko Asai, Hidemichi Watari

**Affiliations:** 1 Obstetrics and Gynecology, Hokkaido University Hospital, Sapporo, JPN; 2 Obstetrics and Gynecology, Ebetsu City Hospital, Ebetsu, JPN

**Keywords:** beckwith-wiedemann syndrome, biomarker, cell-free dna, fetal fraction, fragment size

## Abstract

Beckwith-Wiedemann syndrome (BWS) is a condition present from birth that involves excessive growth and is linked to changes in specific genes located on chromosome 11p15.5. Prenatal diagnosis is mainly based on imaging findings such as macrosomia, macroglossia, and omphalocele, but detection remains difficult. We report a case of a fetus suspected of having BWS based on prenatal ultrasound and MRI. A female infant was delivered via cesarean section at 37 weeks and one day of gestation, showing macrosomia, macroglossia, and other clinical features consistent with BWS. To explore potential biomarkers for prenatal diagnosis of BWS, maternal blood was collected at 36 and 37 weeks of gestation and postpartum days 1 and 5. Cell-free DNA (cfDNA) analysis revealed a bimodal fragment size distribution with peaks at 144 and 166 bp during pregnancy. After delivery, the 144 bp peak disappeared, resulting in a unimodal pattern. The fetal fraction was elevated during pregnancy (33.9-34.5%) and decreased rapidly postpartum (to 3.4%). These findings suggest an increased release of fetal-derived cfDNA with BWS-affected fetuses. This case highlights the potential utility of cfDNA analysis as a noninvasive biomarker for BWS.

## Introduction

Beckwith-Wiedemann syndrome (BWS) is a congenital overgrowth disorder caused by genetic and epigenetic abnormalities at chromosome 11p15.5 [1]. Recently, assisted reproductive technologies such as in vitro fertilization have drawn attention to potentially increasing the risk of imprinting disorders, including BWS [2]. BWS presents with diverse symptoms, typically including macroglossia, omphalocele, neonatal hypoglycemia, and hemihyperplasia [3]. Due to risks such as birth trauma from overgrowth, neonatal airway obstruction from macroglossia, and hypoglycemia, prompt medical intervention is often needed [4]. Additionally, affected individuals have a high risk of developing embryonal tumors like Wilms tumor and hepatoblastoma, especially within the first two years of life, requiring careful follow-up [5]. Prenatal diagnosis is essential for effective perinatal management [6].

Prenatal diagnosis of BWS is mainly based on ultrasound and fetal MRI findings [7-10]. However, in mild cases without omphalocele, imaging-based prenatal diagnosis remains limited [1,11], with a reported detection rate of only 25.5-39.9% [12,13]. Despite advancements in imaging techniques, diagnostic rates have not significantly improved [13]. Although maternal serum markers such as alpha-fetoprotein (AFP) and human chorionic gonadotropin (hCG) have been investigated, their clinical utility remains limited due to low specificity and inconsistent results. For example, elevated AFP levels have been reported in some BWS cases due to omphalocele or placentomegaly, but such findings are not exclusive to BWS and can also occur in other conditions such as neural tube defects or abdominal wall anomalies [14]. Similarly, increased hCG levels lack disease specificity and show considerable variability among affected pregnancies [15]. Therefore, these markers are insufficient for reliable prenatal diagnosis of BWS.

Cell-free DNA (cfDNA) in maternal plasma - small DNA fragments released into the bloodstream from both maternal and fetal cells - has been applied as a noninvasive prenatal testing tool for the detection of chromosomal aneuploidies. Recent advances in sequencing technologies have further enabled its use in identifying single-gene disorders such as achondroplasia, Noonan syndrome, and β-thalassemia, and research into its broader clinical utility is increasingly progressing. Building on this progress, cfDNA may also be utilized as a tool for the prenatal diagnosis of epigenetic syndromes such as BWS. In particular, analysis of fetal fraction and cfDNA fragment size may offer new insights into the diagnosis of BWS.

In this report, we analyzed cfDNA from a pregnant woman carrying a fetus suspected of having BWS. We focused on fragment size distribution and fetal fraction of cfDNA, aiming to explore the characteristics of cfDNA in BWS and its potential utility in prenatal diagnosis.

## Case presentation

The patient was a 26-year-old primigravida who conceived naturally. She had no underlying health conditions or family history of genetic disorders. Increased amniotic fluid volume was noted from 13 weeks of gestation, and polyhydramnios (amniotic fluid index (AFI) of 28 cm) was diagnosed at 25 weeks. At 30 weeks, she was referred for further evaluation due to suspected polyhydramnios and fetal nephromegaly. The estimated fetal weight was 2,346 g (+4.4 SD), AFI was 34 cm, and bilateral nephromegaly was observed. No omphalocele was identified.

**Figure 1:**
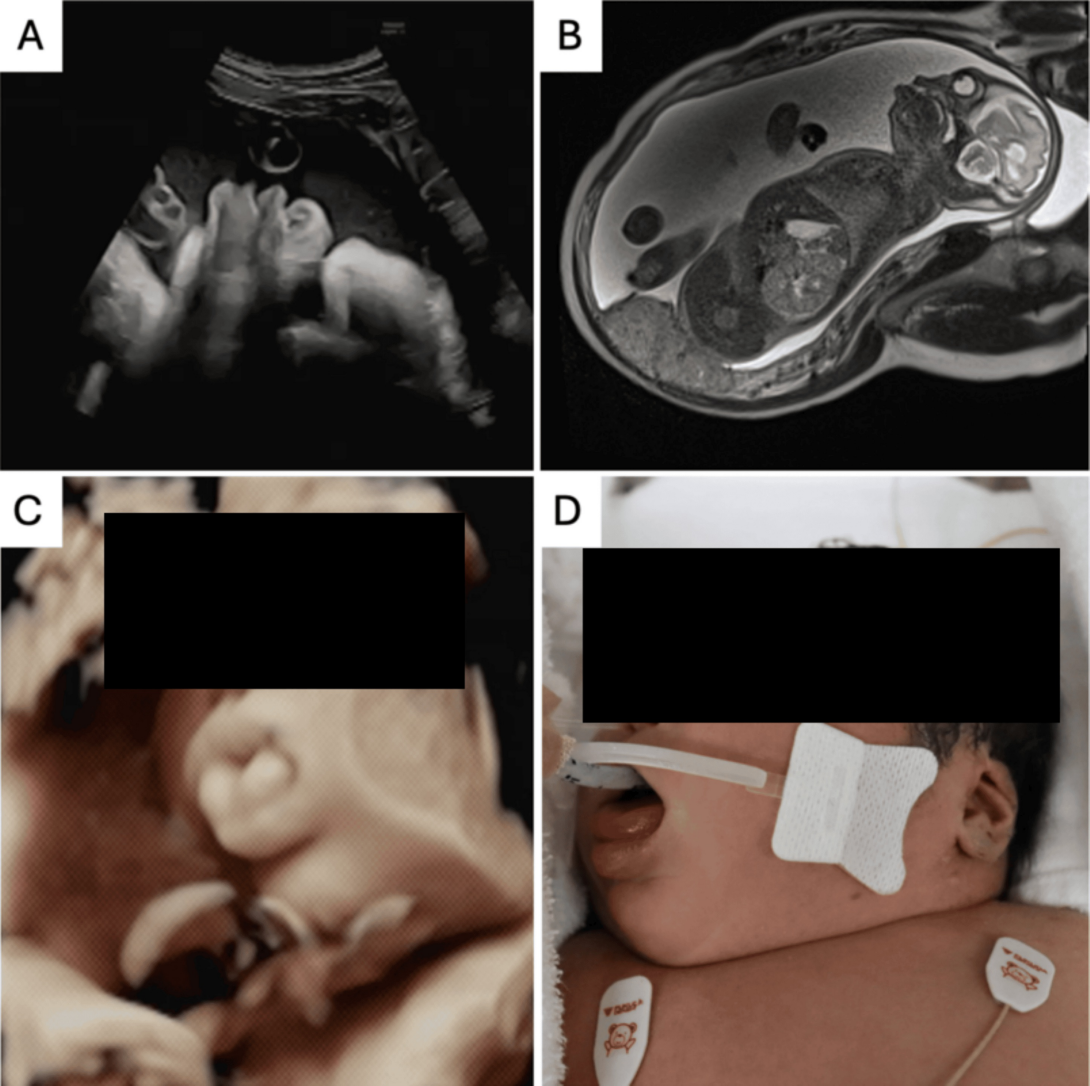
Perinatal findings of the fetus affected by BWS (A) Transabdominal ultrasound image at 31 weeks of gestation showing fetal macroglossia. (B) Fetal MRI at 31 weeks of gestation revealed bilateral nephromegaly, macroglossia, and polyhydramnios. (C) A 3D ultrasound image at 35 weeks of gestation demonstrated the fetal tongue protruding outside the oral cavity. (D) Postnatal photograph of the neonate showing macroglossia, consistent with the clinical feature of BWS. BWS, Beckwith-Wiedemann syndrome

At 31 weeks, an abdominal ultrasound revealed macroglossia, raising the suspicion of BWS. At 35 weeks, a 3D ultrasound clearly showed the tongue protruding from the mouth (Figure 1). At 36 weeks, bilateral nephromegaly, macroglossia, and polyhydramnios were confirmed by MRI. As no other differential diagnoses were evident, the likelihood of BWS increased. At 37 weeks, maternal serum AFP was 1,910.5 ng/mL (reference range in third trimester: approximately 100-500 ng/mL) and hCG was 164,864.0 mIU/mL (reference range in third trimester: approximately 3,000-90,000 mIU/mL), both elevated biomarkers consistent with BWS characteristics.

At 37 weeks and one day of gestation, an elective cesarean section was performed, and a female infant was delivered. Birth weight was 5,364 g (+8.2 SD). The Apgar scores were 1 at one minute and 8 at five minutes. Umbilical arterial blood pH was 7.26 (reference range: 7.20-7.30). The newborn had airway obstruction due to macroglossia and required intubation and neonatal intensive care. Clinical findings included macrosomia, macroglossia, linear ear lobe creases, nephromegaly, and hypoglycemia. The placenta weighed 1830 g, approximately three times the normal weight.

Based on the international consensus diagnostic criteria for the Beckwith-Wiedemann spectrum [16], the patient was evaluated using the recommended clinical scoring system. The case presented two cardinal features - macroglossia and neonatal hypoglycemia, each contributing 2 points - and four suggestive features - macrosomia, nephromegaly, polyhydramnios, and ear creases, each contributing 1 point. The total score was 8 points, exceeding the diagnostic threshold of 4 points required for a clinical diagnosis of classical BWS. Therefore, the diagnosis of BWS was made based on clinical criteria even in the absence of molecular confirmation.

Investigation of maternal cfDNA

To explore potential biomarkers for prenatal diagnosis of BWS, maternal peripheral blood was collected at four time points: 36 weeks zero days, 37 weeks zero days of gestation, postpartum day 1, and postpartum day 5. Blood samples were collected into Cell-Free DNA BCT tubes (Streck, La Vista, NE, USA), and after collection, samples were processed by a two-step centrifugation protocol to separate plasma. cfDNA was then extracted from the plasma using the QuickGene cfDNA Isolation Kit (catalog no. CF-L; Kurabo, Osaka, Japan). With informed consent, cfDNA concentration, fragment size distribution, and fetal fraction were analyzed. The concentration and size distribution of cfDNA were measured using the 4150 TapeStation system (Agilent Technologies, Santa Clara, CA, USA), and fetal fraction was measured by GeneTech Inc. (Tokyo, Japan).

**Figure 2:**
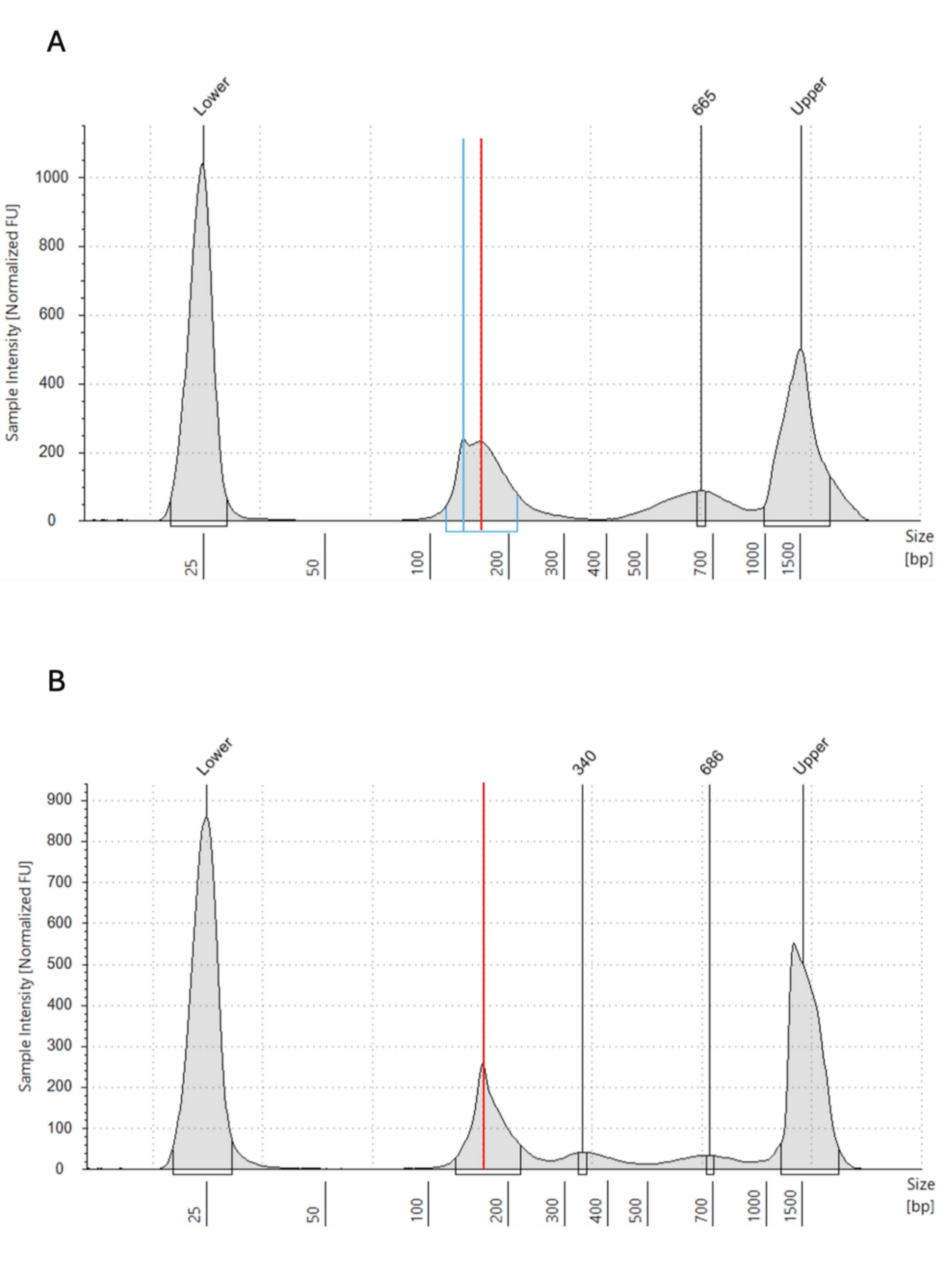
Comparison of fragment size of maternal cfDNA before and after delivery (A) At 36 weeks zero days of gestation, maternal plasma cfDNA exhibited a bimodal distribution with two peaks at approximately 144 bp (blue line) and 166 bp (red line). (B) After delivery, the 144 bp peak disappeared, resulting in a unimodal distribution with a predominant peak at 166 bp (red line). cfDNA, cell-free DNA

cfDNA concentrations were 168 ng/mL at 36 weeks, 161 ng/mL at 37 weeks, 115 ng/mL on postpartum day 1, and 97 ng/mL on postpartum day 5, decreasing after delivery. During pregnancy (36 and 37 weeks), the cfDNA fragment size distribution showed a bimodal pattern with peaks at 144 bp and 166 bp. Postpartum samples showed a unimodal distribution with a single peak at 166 bp (Figure 2).

**Figure 3:**
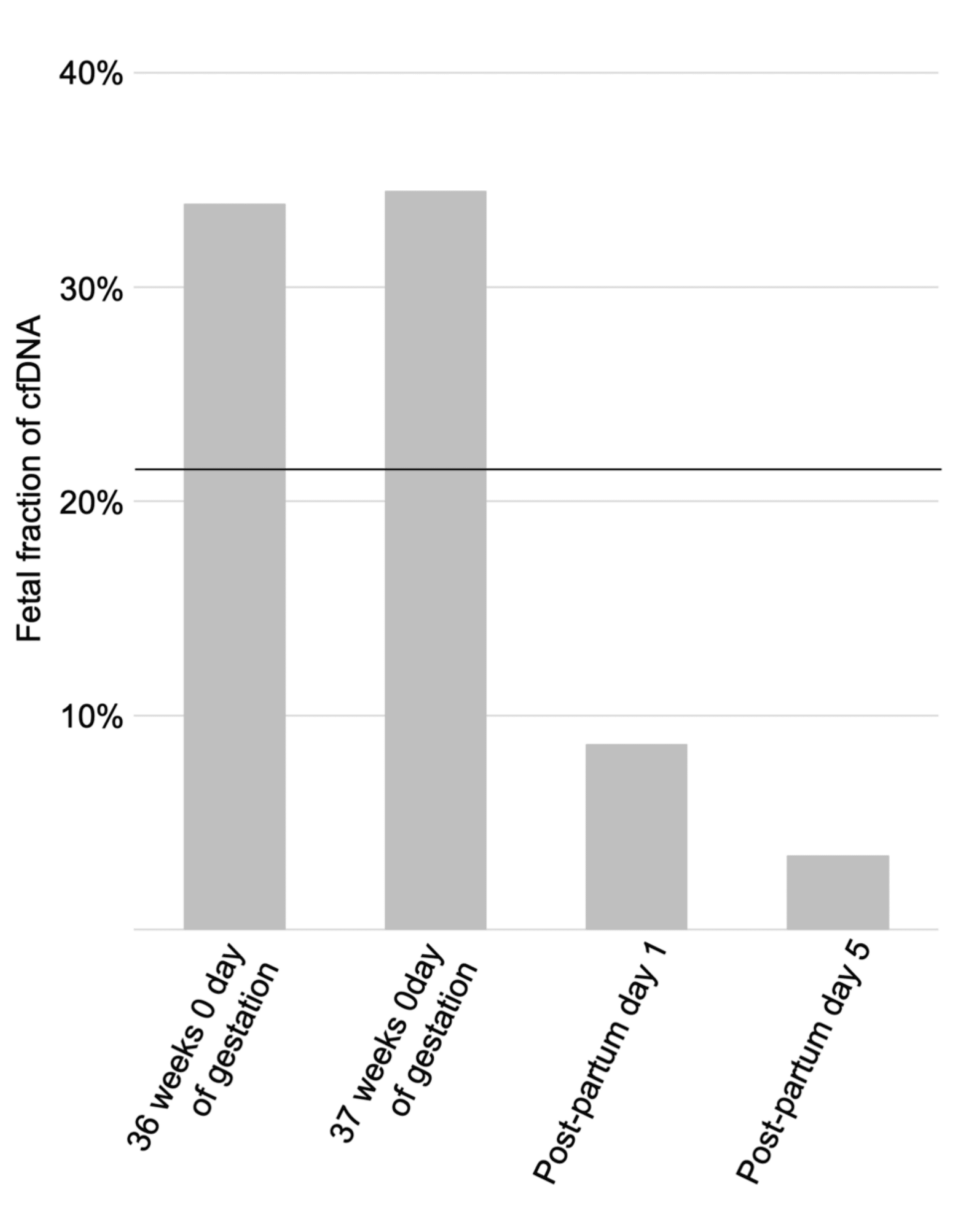
Changes in fetal fraction of maternal plasma cfDNA before and after delivery cfDNA, cell-free DNA

The fetal fraction was 33.9% at 36 weeks, 34.5% at 37 weeks, 8.6% on postpartum day 1, and 3.4% on postpartum day 5, decreasing rapidly after birth. The fetal fraction before delivery was higher than the average of 21.17% observed in healthy pregnancies after 29 weeks of gestation [17], suggesting an increased release of fetal-derived cfDNA into the maternal bloodstream in BWS (Figure 3). 

## Discussion

This case provides new insights into maternal cfDNA characteristics during pregnancy with a BWS-affected fetus. Notably, a bimodal cfDNA fragment size distribution was observed during pregnancy, which shifted to a unimodal distribution postpartum. Additionally, the fetal fraction during pregnancy was markedly higher than in normal pregnancies.

Typically, fetal-derived cfDNA fragments are approximately 144 bp, while maternal-derived cfDNA fragments are about 166 bp [18]. In this case, the bimodal peaks at 144 bp and 166 bp before delivery suggest a high abundance of fetal cfDNA, while the disappearance of the 144 bp peak postpartum indicates a shift to predominantly maternal cfDNA. This observation aligns with our previous study, which estimated the half-life of cfDNA to be approximately 24.2 minutes, explaining the rapid change in distribution after delivery [19].

Furthermore, the fetal fraction was significantly elevated at 34% during pregnancy. Hou et al. reported a mean fetal fraction of 21.17% after 29 weeks in normal pregnancies [17], indicating that the value in this case was exceptionally high. Fetuses with BWS are known to exhibit overgrowth and placental enlargement, which may lead to increased release of fetal cfDNA into the maternal circulation. This could explain both the bimodal fragment size distribution and the elevated fetal fraction.

The precise biological mechanisms underlying the increased release of fetal-derived cfDNA in BWS remain unclear. However, BWS is characterized by fetal overgrowth, placentomegaly, and organomegaly, all of which may contribute to increased cell turnover or apoptosis in fetal and placental tissues. These processes could result in the elevated release of cfDNA into the maternal circulation. Additionally, abnormal epigenetic regulation at 11p15.5 may lead not only to overexpression of growth-promoting genes such as IGF2 but also to dysregulation of cellular proliferation and death pathways. These pathophysiological changes may underlie the observed increase in cfDNA levels and altered fragment size profiles. Further studies are needed to elucidate the molecular mechanisms connecting BWS-related genetic and epigenetic alterations with cfDNA dynamics.

The observed marked elevation in the fetal fraction and the bimodal fragment size distribution during pregnancy, in this case, suggest that pregnancies with BWS-affected fetuses may show a higher proportion of fetal-derived cfDNA in maternal blood, highlighting the potential of cfDNA analysis for prenatal diagnosis. A higher fetal fraction may improve the sensitivity and specificity of genetic and epigenetic testing using cfDNA [20]. Currently, prenatal diagnosis of BWS relies heavily on imaging techniques. However, in the future, cfDNA-based genetic and epigenetic testing may provide a definitive diagnosis noninvasively.

However, this report describes only a single case, and therefore, the generalizability of these findings is limited. To validate the association between BWS and characteristic cfDNA patterns such as elevated fetal fraction and bimodal fragment size distribution, larger studies involving multiple cases are needed. Future research may clarify whether these cfDNA characteristics are consistently observed in BWS and whether they can be reliably used for diagnostic purposes. If confirmed, cfDNA-based analysis could be incorporated into clinical diagnostic protocols, offering a noninvasive and potentially earlier tool for the prenatal detection and management of BWS.

While our study focused on the analysis of cfDNA fragment size and fetal fraction, it did not investigate specific genetic or epigenetic alterations within cfDNA that are directly associated with BWS. BWS is primarily caused by imprinting defects and mutations in genes located at chromosome 11p15.5, such as CDKN1C, H19, and IGF2 [1]. Future studies incorporating high-throughput sequencing or methylation analysis of cfDNA may help identify disease-specific molecular signatures, thereby improving the diagnostic accuracy and enabling earlier noninvasive prenatal diagnosis of BWS.

## Conclusions

This case demonstrates that during pregnancy with a fetus affected by BWS, maternal cfDNA shows a bimodal fragment size distribution and an elevated fetal fraction. These findings suggest a substantial increase in fetal-derived cfDNA in maternal blood. With future advancements in molecular diagnostic techniques, analysis of maternal cfDNA may become a key tool for the prenatal diagnosis of BWS, warranting further research in this area.
